# Granulomatous polyangiitis involving the fourth ventricle: Report of a rare case and a literature review

**DOI:** 10.1515/biol-2022-0654

**Published:** 2023-07-17

**Authors:** Dan Yuan, Qing Ji, Jin-Hua Xia, Jin-Jing Wang, Na Liang

**Affiliations:** Department of Pathology the Affiliated Hospital of Zunyi Medical University, Zunyi 563000, Guizhou, China; Department of Histology and Embryology, Zunyi Medical University, Zunyi, Guizhou 563000, China

**Keywords:** granulomatous polyangiitis, Wegener’s granulomatosis, fourth ventricle, necrotizing granulomatous vasculitis, treatment

## Abstract

Granulomatous polyangiitis (GPA) is a rare systemic autoimmune vasculitis disease that is highly correlated with anti-neutrophil cytoplasmic antibodies (ANCAs). It was formerly called as “Wegener’s granulomatosis.” The clinical manifestations are diverse, mainly involving the upper respiratory tract, lungs, and kidneys, and this disease can involve the brain parenchyma as an isolated solid mass. Only one case has been reported thus far. To provide further information on this rare case, we report a case of GPA involving the fourth ventricle and review the relevant literature. A 32-year-old Chinese female developed fever, cough, and shortness of breath for 20 days. An 80 mm × 80 mm skin ulcer was seen on the right lower limb. CT showed multiple large patches of increased density in both lungs. The patient’s serological ANCA was positive. Later, the patient developed dizziness and headache. Magnetic resonance imaging of the head showed a mass of approximately 21 mm × 24 mm in the fourth ventricle. The patient had a craniotomy for mass resection, and macroscopically, the mass was gray–red and measured 25 mm × 20 mm × 20 mm, was soft, had local hemorrhage and necrosis, and had no capsule. The main microscopic features included necrotizing granulomatous vasculitis, the patient’s immunohistochemistry was positive for CD68 and negative for glial fibrillary acidic protein, and the acid-fast staining and hexaamine silver staining were negative. Combined with the clinical history, serology, and imaging, the pathological diagnosis was GPA in the fourth ventricle. The patient was switched to rituximab combined with steroid therapy because she did not tolerate cyclophosphamide. After 5 months of follow-up, the patient’s lung lesions and skin ulcers had completely improved, but the brain lesions had further progressed. When a patient has multiple system diseases, abnormal clinical manifestations, and positive serological ANCAs, a diagnosis of GPA should be carefully considered, and biopsies of easy-to-access sites should be performed. If the patient’s histopathological manifestations include vasculitis, granuloma, and necrosis, a diagnosis of GPA is more likely. If a patient subsequently develops an intraventricular mass, the clinicians should consider a diagnosis of GPA, which can rarely involve the cerebral ventricle to avoid an unnecessary biopsy or surgical treatment of intracranial lesions. When a patient is intolerant to the traditional treatment drug cyclophosphamide and needs to be switched to rituximab, the treatment effect of intracerebral lesions is not ideal; therefore, the treatment of lesions involving GPA in the ventricle is worthy of further exploration.

## 1 Background

Granulomatous polyangiitis (GPA), formerly known as “Wegener’s granulomatosis (WG),” is an anti-neutrophil cytoplasmic antibody (ANCA)-related vasculitis, which is a type of autoimmune systemic small and medium vasculitis that is characterized by the production of pathogenic ANCAs [1]. GPA is a disease with an incidence rate of 3/100,000 [2], and it is more prevalent in patients of European descent. Its incidence appears to increase with distance from the equator, and there is no difference between male and female incidence rates [22]. It mainly affects the upper respiratory tract, lungs, and kidneys. The clinical manifestations include systemic necrotizing small vasculitis, necrotizing granulomatous inflammation, and glomerular necrotizing nephritis. Before the advent of therapeutic drugs (prednisone and cyclophosphamide [CYC]), the rate of nervous system involvement was 25.7–54%, and after the emergence of effective therapeutic drugs, the rate of nervous system involvement has decreased to 22% [3–5]. The nervous system involvement mainly manifests as meningitis and cerebrovascular events, while isolated solid mass lesions in the brain parenchyma are rare [6,7,17]. The GPA pathology mainly includes a necrotizing granulomatous vasculitis of the small and medium blood vessels. The diagnosis of GPA needs to be combined with the clinical history and serological, radiological and pathological findings, and histopathology is the gold standard for diagnosing GPA [8,9]. GPA cases involving the fourth ventricle are rare, and there is only one related report that was found in PubMed. Therefore, this article explains the clinicopathological analysis, diagnosis, differential diagnosis, and treatment of a case of GPA involving the fourth ventricle, and this article aims to improve the clinicians’ and pathologists’ knowledge of this disease.

## 2 Case characteristics

**Figure 1:**
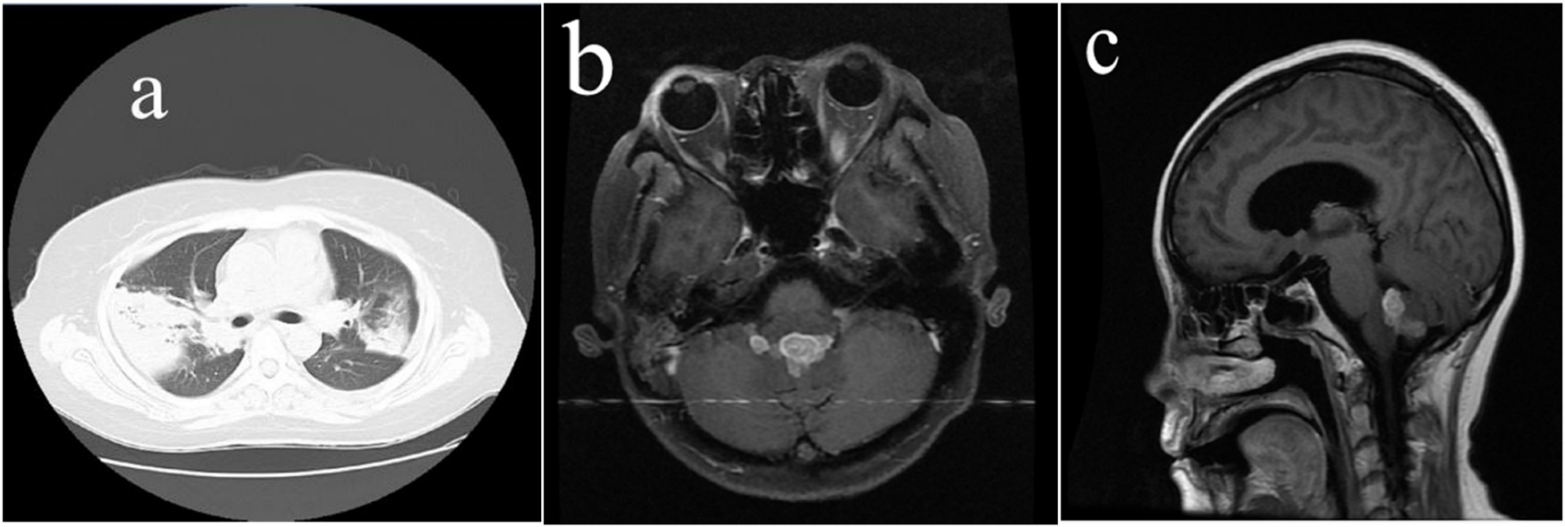
(a) Chest CT showing large patches and a patchy increased density in both lungs, and multiple bronchial stenoses in both lungs. (b and c) Head MRI showing irregular masses in the fourth ventricle, with a larger cross-section of approximately 21 mm × 24 mm, spreading along the bilateral lateral foramen of the fourth ventricle, and the sinus bone and surrounding pia mater were intact.

**Figure 2:**
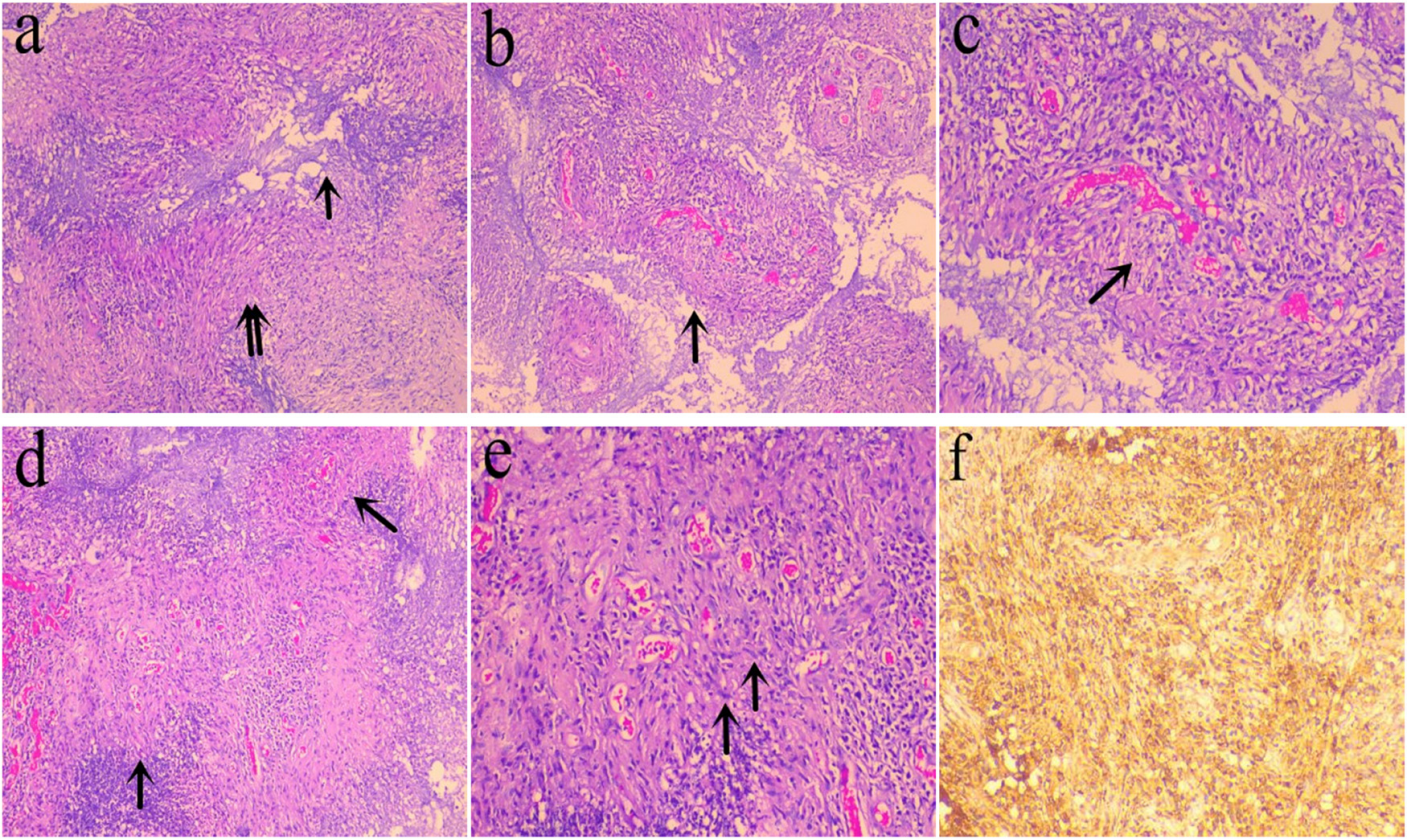
Histopathology and immunohistochemistry: histopathology: (a) There was an abscess formed by liquefaction necrosis, the epithelioid cells around the abscess were arranged in a fence to form granulomas, and a large number of inflammatory cells infiltrated in the interstitium (single arrow abscess, double arrow granulomas, ×100). (b and c) Vasculitis: small blood vessels infiltration of plasma cells, lymphocytes, and neutrophils into the wall, fibrinoid necrosis is not obvious (×100) (single arrow vasculitis, ×200). (d and e) A large number of epithelioid cells around the blood vessels form a granulomatous vasculitis (d: single arrow granulomatous vasculitis, ×100; e: single arrow epithelioid cells, ×200). Immunohistochemistry: (f) there was positive CD68 expression in epithelioid cells (×100).

A 32-year-old Chinese female patient was admitted to the hospital with “fever, cough, and shortness of breath for 20 days.” A chest CT that was performed at the local hospital indicated tuberculosis, and the lesions increased in size after anti-tuberculosis treatment. Later, the patient went to our hospital. The patient’s physical examination showed that she had wheezing that could be heard and was scattered in both lower lungs, and 80 mm × 80 mm skin ulcers were seen on her right lower extremity. An auxiliary examination included a chest CT, which showed large patches, patchy increased densities in both lungs, and multiple bronchial stenoses in both of her lungs (Figure 1a). Her ANCA was positive, Anti-protease 3 (anti-PR3) antibody was increased; she had anemia and high white blood cell and platelet counts. She also had increased C-reactive protein, erythrocyte sedimentation rate, anti-nuclear antibody spectrum, and anti-cyclic citrullinated peptides. The level of antibody was normal. The patient’s urine protein was 2+, and urine occult blood was 3+. Multiple sputum and alveolar lavage samples were negative for tuberculosis bacteria; the patient’s clinical diagnosis was GPA. For her treatment, she was given methylprednisolone sodium succinate combined with CYC for anti-inflammatory and immune suppression. Because the patient was intolerant to CYC and experienced severe nausea and vomiting, she was treated with rituximab (RTX) combined with steroids. After 1 year, the patient developed new symptoms such as dizziness and headache. A cranial MRI showed that she had an irregular mass that was seen in the fourth ventricle, with a larger cross-section of approximately 21 mm × 24 mm, which spread along the bilateral lateral foramen of the fourth ventricle. The sinus bone and surrounding pia mater were intact, and tumorous lesions were suspected (Figure 1b and c). The clinician performed a craniotomy to excise the mass. During the operation, a gray and red mass in the fourth ventricle (25 mm × 20 mm × 20 mm in size, with an abundant blood supply and a tight adhesion with the surrounding tissues, invading the dorsal side of the brainstem) was removed and was submitted for a pathological evaluation. Macroscopy revealed a gray–red mass that measured 25 mm × 20 mm × 20 mm, was soft, and had local bleeding and necrosis but no capsule. The microscopic features included vasculitis, granuloma, and necrosis, manifested by the infiltration of plasma cells, lymphocytes, and neutrophils in the walls of arterioles, venules, and capillaries. The presence of fibrinoid necrosis was not obvious (Figure 2b and c), and a large number of epithelioid cells were around the blood vessels. Surrounding the formation of granulomatous vasculitis (Figure 2d and e), the necrosis included liquefaction necrosis that formed abscesses. The epithelioid cells around the abscesses were arranged in a fence to form granulomas, and there were a large number of neutrophils, lymphocytes, tissue cells, and a small amount of plasma cell infiltration (Figure 2a). Immunohistochemistry was positive for CD68 (Figure 2f) and was negative for glial fibrillary acidic protein (GFAP). Acid-fast staining and silver hexaamine staining were negative. After combining the patient’s clinical history, serology, imaging, pathological diagnosis, she was diagnosed with fourth ventricle GPA. After 5 months of follow-up, the patient’s lung lesions and skin ulcers had completely resolved, but her brain lesions had further progressed (clinical data is shown in Table 1).

**Table 1:** Clinical data of two cases of ventricular GPA

Case	Age (years)	Gender	Position	Symptom	Pathologic diagnosis	Treatment and prognosis
1. Berlis [17]	57	Male	Fourth ventricle	Difficulty breathing, bloody rhinitis, and hoarseness, followed by nausea, vomiting, and headache	GPA of fourth ventricle	High dose steroids combined with CYC. The patient died 4 months later
2. (This report)	32	Female	Fourth ventricle	Fever, cough, shortness of breath, followed by dizziness and headache	GPA of fourth ventricle	Large amounts of steroid hormones combined with RTX monoclonal antibody treatment. Followed up for 5 months, further development of brain lesions


**Informed consent:** Informed consent has been obtained from all individuals included in this study.
**Ethical approval:** The research related to human use has been complied with all the relevant national regulations, institutional policies and in accordance with the tenets of the Helsinki Declaration, and has been approved by the authors’ institutional review board or equivalent committee.

## 3 Discussion

GPA is a kind of ANCA-associated vasculitis. The other types of ANCA-related vasculitis also include microscopic polyangiitis and eosinophilic GPA. These diseases are all related to circulating ANCAs, and their main target antigen is protease 3 (PR3). Myeloperoxidase (MPO) and GPA are mainly related to PR3-ANCA (75%) [1]. The specific pathogenesis may be the interaction of immune, infection, or genetic factors that cause PR3. The increase and release of MPO expression leads to the production and proliferation of pathogenic ANCAs. ANCAs combine with PR3 and MPO to form ANCA-PR3 and ANCA-MPO immune complexes and activate neutrophils, and the activated neutrophils pass through blood vessel walls. After a series of reactions, such as with respiratory bursts and degranulation, ANCAs lead to the damage of the vascular endothelial cells, causing vasculitis and necrosis and then the accumulation of monocytes to induce granulomatous inflammation [10,11]. The pathogenesis of GPA in the brain may be due to [12,13]: (1) inflammation, blockage, or an increased permeability of small and medium blood vessels in the brain caused by systemic vasculitis; (2) the infiltration or compression of granulomatous lesions in adjacent structures; and (3) the development of new granulomatous lesions in the central nervous system. Generally, lesions involving the dura mater or pituitary gland are mainly attributed to the infiltration of granulomas in adjacent structures, while parenchymal lesions are mediated by vasculitis and a destruction of the blood–brain barrier [14]. In this case, the GPA involved the fourth ventricle. CT showed that the sinus bone and surrounding pia mater were intact; therefore, the direct spread of adjacent granulomas was rule out. The pathogenesis may be related to vasculitis and the destruction of the blood–brain barrier. GPA can affect all systems throughout the body [14,15], most commonly the ears, nose and throat, lungs, and kidneys, and can also affect the skin, orbits and eyes, gastrointestinal tract, breasts, cardiovascular, peripheral nerves, and central nervous system. The symptoms of GPA that involve the central nervous system vary depending on the specific structures involved (dura mater, brain parenchyma, pituitary, spinal cord, and pia mater), and the symptoms can manifest as headache, intracranial hemorrhage, cerebral infarction, and encephalopathy (epilepsy, changes in consciousness, and neuropsychiatry). De Luna et al. [16] studied 35 patients who had GPA involving the central nervous system. Headache was the main symptom, followed by sensory abnormalities and dyskinesias. Central nervous system involvement included 20 cases of dura meningitis, 15 cases of cerebral ischemic lesions, and hemorrhagic lesions. The pituitary gland was involved in two cases, indicating that CPA involvement of the central nervous system was mainly manifested as dura materitis or cerebrovascular events [16], while the fourth ventricle involving the brain parenchyma was rarely seen with a mass. Only one report was found in the literature. Berlis [17] reported a 57-year-old man who complained of dyspnea, bloody rhinitis, and hoarseness. ANCAs specific to PR3 were detected in that patient’s serum, a suspicious right lung tumor was found by chest radiology, and histopathological examination after right upper lobe resection confirmed the diagnosis of GPA. Steroids and CYC were given as immunosuppressive therapy. Secondary to the ineffectiveness or from the side effects of the immunosuppressive regimen, the patient complained of nausea, vomiting, and headache, as well as progressive neurological deficits, deflection, mild nystagmus, and diplopia. Magnetic resonance imaging revealed a clear mass in the fourth ventricle, which squeezed and infiltrated the surrounding structures. After treatment with high-dose steroids and CYC, the patient’s neurological symptoms improved. MRI showed that the fourth ventricle mass had subsided. Although the mass had subsided, the patient’s lung disease was still progressing. The patient died 4 months later [17] (clinical data is shown in Table 1). In our case, the patient complained of fever, cough, shortness of breath, bilateral lung disease, right lower limb skin ulcer, and had other multiple system involvement manifestations and a positive serum ANCA. The clinical diagnosis of GPA was based on the response to steroids and RTX as immunosuppressive therapy, and then the patient developed dizziness and headache, and irregular masses in the fourth ventricle, which were seen on the cranial MRI. These lesions were suspected to be tumorous lesions. A grayish red tumor in the fourth ventricle was seen during the operation. The tumor was removed and sent to pathology. The pathological diagnosis confirmed GPA. The diseases that need to be considered include neoplastic lesions (such as glioblastoma and ependymoma) and infectious lesions (such as tuberculosis). The epithelioid cells that are arranged around blood vessels are similar to the vascular chrysanthemum in ependymoma. Ependymoma, where the epithelioid cells are arranged in a fence around the necrosis, could indicate glioblastoma, but the necrosis is inflammatory necrosis, which is when a large number of inflammatory cells infiltrate in the interstitium, the cells are not abnormal, no mitotic figures are seen, and GFAP, glioblastoma, and ependymoma are excluded. No typical caseous necrosis was seen in the lesion, and acid-fast staining and hexaamine silver staining were negative to exclude tuberculosis and other special infections. The treatment of GPA is divided into two stages, namely, the induction of remission and the maintenance of remission. For GPA patients who have central nervous system involvement, the remission induction therapy mainly includes high-dose steroids and CYC. Patients with CYC intolerance can be switched to RTX; once a complete remission is achieved, patients should be switched to maintenance therapy. In the treatment program, a combination of low-dose steroids and oral immunosuppressive agents, including methotrexate, mycophenolate mofetil, azathioprine, or RTX, should be used for at least 24 months [13]. If there is diffuse alveolar hemorrhage or if the serum creatinine level is ≥500 μmol/L, plasma exchange should be considered [9]. According to reports, two-thirds of patients with GPA involving the pituitary gland with conventional remission induction therapy (high-dose glucocorticoid and oral or intravenous CYC) can obtain relief [18]. Cartin’s [19] studies have shown that patients with GPA treated with RTX achieve complete remission faster than those treated with CYC, and this treatment may be superior in terms of preventing recurrent disease. These findings suggest that clinicians may consider using RTX as a first-line treatment [20]. However, there is limited experience in using RTX in treating the pituitary involvement in GPA, and further research is needed [20,21]. This patient was mainly treated with a large number of steroid hormones combined with RTX (due to CYC intolerance). The patient’s lung lesions were significantly improved during the 5-month follow-up, but the patient’s brain lesions were still progressing, as reported by Berlis. The outcome of this patient after RTX was the opposite (the brain lesions improved, and the lung lesions further progressed), and this case was not completely consistent with the report of Cartin. This analysis may be compared with CYC. The rate of blood–brain barrier passage of RTX is low, and a lower brain drug concentration is related to not achieving a good therapeutic effect. Therefore, the treatment of intracerebral lesions in patients with GPA involving the fourth ventricle and who have an intolerance to CYC is worthy of further exploration. We can change the mode of RTX administration from intravenous to intrathecal injection. Intrathecal injections typically do not need to pass through the blood–brain barrier because the drug diffuses directly into the ventricle via cerebrospinal fluid; however, the administration time and concentration are unknown and require further research. Spleen tyrosine kinase (SYK) and Bruton tyrosine kinase (BTK) are other potential therapeutic targets for GPA. SYK is a non-receptor tyrosine kinase that reduces ANCA-induced cellular responses by mediating the signaling of B cell receptors, Fc receptors, integrins, and pattern recognition. BTK mediates B cell receptor signaling, influencing growth and maturation [22]. According to research, untreated GPA patients have an average life expectancy of 5 months and a 1-year survival rate of less than 30%. Recently, over 80% of patients who receive effective treatment can still expect to live at least 8–9 years [1].

## 4 Conclusion

When the patient has multiple systemic diseases (especially involving the upper respiratory tract, lungs, and kidneys), abnormal clinical manifestations, and positive serological ANCAs, a diagnosis of GPA should be carefully considered, and a biopsy of the easy-to-access parts should be performed. If the histopathological findings include a necrotizing granulomatous vasculitis, the diagnosis of GPA is more likely. If the patient subsequently develops an intraventricular mass, clinicians should be alert to the possibility of the rare manifestation of GPA involving the cerebral ventricle to avoid an unnecessary biopsy or unnecessary surgical treatment of intracranial lesions. When patients are intolerant to the traditional treatment drug CYC and need to switch to RTX, the treatment effect of intracerebral lesions is not ideal, so the treatment of lesions involving GPA in the ventricle is worthy of further exploration.
